# Engineering fratricide-resistant CCR4/CD7 CAR T cells with enhanced safety and persistence for T cell malignancies

**DOI:** 10.1016/j.omton.2026.201193

**Published:** 2026-04-20

**Authors:** Sile Li, Wenwei Tu, Wing Leung

**Affiliations:** 1 Department of Paediatrics and Adolescent Medicine, Li Ka Shing Faculty of Medicine, The University of Hong Kong, Hong Kong SAR, P.R. China; 2 Children’s Blood and Cancer Centre, KK Women’s and Children’s Hospital, SingHealth Duke-NUS, Singapore, Singapore

## Main text

**Figure 1:**
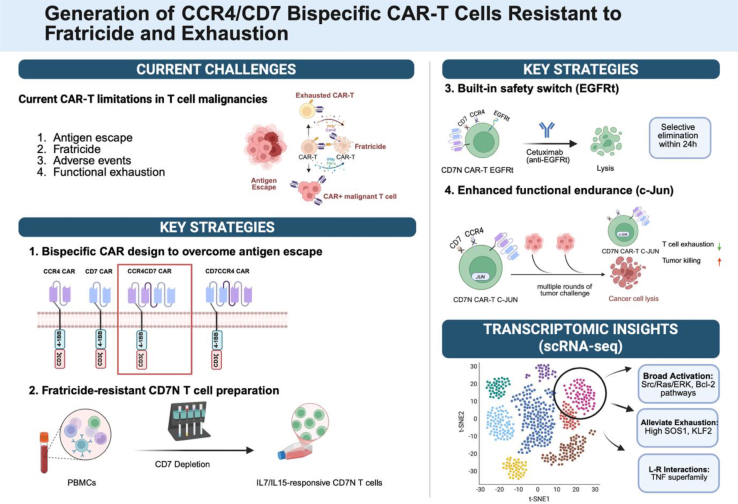
Schematic overview of key challenges in CAR-T therapy for T cell malignancies, with potential solutions demonstrated by Li et al.

Despite the remarkable success of chimeric antigen receptor (CAR)-T cell therapy in B cell malignancies, its application to T-lineage neoplasms has been hampered by antigen escape, fratricide, functional exhaustion, and severe adverse events, such as T cell aplasia, or inadvertent transduction of malignant T cells with the CAR construct (Figure 1).^1^ In a recent study,^2^ we sought to collectively address these limitations through the rational design of a bispecific CAR targeting both CD7 and CCR4 using CD7-negative (CD7N) T cells, combined with strategic functional enhancements and safety elements (Table 1).

**Table 1:** Overview of key findings and potential advantages of CCR4/CD7 bispecific CD7N CAR-T cell engineering

Key problems	Engineering solutions	Potential advantages	Key experimental findings of Li et al.
1. Antigen heterogeneity contributing to antigen escape and tumor resistance	*Bispecific CAR design*: engineering a single CAR construct with two scFvs targeting both CD7 and CCR4. Selecting the optimal tandem configuration (CCR4/CD7).	• Ability to recognize and kill tumor cells expressing heterogeneous levels of CD7 and CCR4. • Broadens therapeutic applicability to both CD7+ immature and CCR4+ mature T cell malignancies. • Ability to kill CCR4+ T regulatory cells. • Broadens therapeutic applicability to both CD7+ AML and CD7+ nonhematopoietic solid malignancies. • Mitigates the risk of disease relapse due to antigen loss.	• CD7N CCR4/CD7 bispecific CAR-T cells showed superior cytotoxicity compared to single-targeting CARs against malignant cells with heterogeneous CD7 and CCR4 expression. • Efficacy against both CCRF-CEM (CD7+/CCR4+) and Jurkat cancer cells (CD7+/CCR4low) *in vivo*.
2. Fratricide and product contamination	*CD7 depletion*: development of a simple single-step immunomagnetic procedure to remove CD7-positive cells before CAR-T production.	• Depletion of normal and malignant T cells simultaneously. • Prevents fratricide during manufacturing and CAR-T cell expansion. • Prevents inadvertent editing or transduction of malignant T cells. • Simple, single-step process that avoids the cost and complexity of genetic manipulations, ensuring genome safety. • No need for additional depletion of CCR4.	• After single-step CD7-depletion, >85% of the resulting CD7N cells were CCR4-negative, indicating further CCR4 depletion is unnecessary. • CD7N cells and CD7N CAR -T cells expanded robustly with IL-7/IL-15 and maintained a CD7-negative phenotype, confirming the lack of fratricide during cell culture.
3. Severe CAR-T cell-mediated adverse events	*EGFRt safety switch*: incorporating an EGFRt into the CAR construct via a bicistronic design.	• CAR-T cells can be electively eliminated by administering the FDA-approved antibody cetuximab. • Can mitigate the occurrence of serious adverse events such as CAR+ leukemia or prolonged T cell aplasia.	• Cetuximab at concentration as low as 1 μg/mL could effectively and rapidly (within 24 h) lyse the EGFRt-expressing CAR T cells.
4. Functional exhaustion	*c-Jun overexpression*: incorporating the transcription factor c-Jun into the CAR construct.	• Prevents CAR-T cell exhaustion by disrupting immunoregulatory activator protein 1 (AP-1)/interferon regulatory factor (IRF) transcriptional complexes. • Enhances functional endurance, potentially leading to more durable cancer remission.	• c-Jun overexpression significantly reduced the frequency of CAR-T cells exhibiting the LAG-3+ exhausted phenotype. • CD7N CAR-T cells with c-Jun showed superior tumor control compared to those without c-Jun in a repetitive tumor challenge assay *in vitro*.
5. The challenges of discovering novel genome-wide modifiable targets and mechanisms	*single-cellRNA-seq(scRNA-seq)analysis*: profiled CD7N CAR-T cells, bulk CAR-T cells, and non-transduced CD7N cells after tumor exposure.	• Allows identification of CAR-T subsets that are uniquely responsive to tumor challenge. • Reveals specific molecular pathways, ligand-receptor interactions, and key genetic determinants associated with CAR-T function, which could be modulated for further improvement.	• ScRNA-seq analyses showed distinct transcriptomic profiles and pathways enrichment in CD7N CAR-T cells, including cell adhesion, activation, and TNF signaling. • A unique AQP3+ proliferative CD4+ CAR-T cell cluster was identified with high expression of SOS1 and KLF2, suggesting potential new modifiers for augmentation of CAR-T therapy.

### Bispecific CAR design to address tumor antigen heterogeneity

The absence of a universally expressed target antigen remains a major barrier to CAR T therapy for T cell malignancies. Antigen heterogenicity is observed intratumorally and across various T-lineage subtypes. While CD7 is frequently expressed on immature T-cell acute lymphoblastic leukemia (T-ALL) and T-cell lymphoblastic lymphoma (T-LBL), CD7 is typically absent in mature T cell malignancies, such as adult T cell leukemia/lymphoma (ATLL), cutaneous T cell lymphoma (CTCL), and peripheral T cell lymphoma (PTCL).^1^ With anti-CD7 CAR T cells, antigen-negative relapses were commonly seen in T-lymphoblastic neoplasms.^1^ To broaden the disease spectrum of CAR-T therapy and to prevent antigen escape, we chose CCR4 as the second target, as it is expressed broadly among mature T cell malignancies and on many immature T-ALL cases.^3^^,^^4^ As CTCL progressed, a loss of CD7 expression and an increase in CCR4 expression were frequently observed in longitudinal studies.^5^ Targeting the CCR4+ T-regulatory cells in the tumor microenvironment may provide additional benefit to enhance CAR-T efficacy.^1^^,^^4^^,^^6^ Among our CAR constructs, the CCR4/CD7 tandem configuration was chosen based on superior cytotoxicity. Each single-chain variable fragment (scFv) retained independent specificity, enabling effective targeting of a spectrum of antigen-heterogenous cancer cells.

### Preparation of CD7N T cells to avoid fratricide and tumor contamination

One unique challenge for T cell targeting is that the antigens are shared between normal T cells and cancer cells, resulting in CAR-dependent fratricide (e.g., with CD7 CAR) or product contamination when malignant cells are co-enriched with normal T cells during CAR-T manufacturing (e.g., with CD4/CD8 selection).^7^ The use of bispecific CCR4/CD7 CAR may further exacerbate fratricide. Previous studies have used various methods to downregulate the antigens on the CAR-T cells, including genome editing, transient CAR-signaling inhibition, or protein expression blocker (PEBL). The use of genome editing and PEBL, however, may compound the risk of relapse if a malignant cell was inadvertently edited or transduced, resulting in antigen escape. In our study, we established a simple immunomagnetic procedure to yield CD7N T cells resistant to fratricide and to deplete CD7+ malignant cells simultaneously. Notably, the majority of these CD7N cells naturally lacked CCR4 expression, eliminating the need for additional costly depletion steps. When cultured with IL-7 and IL-15, the CD7N cells exhibited robust expansion and maintained responsiveness to CD3/CD28 activation, rendering them suitable for clinical-scale CAR T production.

### Built-in EGFRt switch to enhance safety

Another unique on-target, off-tumor side effect is normal T cell aplasia, which renders CAR-T recipients at high risk of infection if the aplasia is long-lasting. CAR transgene-positive malignancy is another severe adverse event and may arise from insertional mutagenesis in a normal T cell or from inadvertent transduction of a contaminated malignant T cell in the CAR-T product, as mentioned above.^7^^,^^8^ Given the potential for these serious events, we generated a CAR construct with truncated epidermal growth factor receptor epidermal growth factor receptor (EGFRt) domain, which enabled elective CAR T elimination by the anti-EGFR antibody cetuximab when clinically indicated at therapeutically achievable concentrations as low as 1 μg/mL.

### Overexpression of c-Jun to enhance functional endurance

One major concern of using CD7N CAR-T cells is vulnerability to exhaustion and apoptosis, as CD7N T cells are often more terminally differentiated and exhibit reduced persistence and survival potential compared with CD7-positive T cells.^9^ In this regard, we showed that CD7N CAR-T cells displayed functional exhaustion rapidly after repeated challenges with leukemia cells, but overexpression of c-Jun significantly prolonged their functional endurance, with significantly reduced LAG3 expression (from 20% to 30% to <10%).

### Transcriptome analyses to uncover alternatives to improve functional persistence

We then sought to identify alternatives to c-Jun. Single-cell RNA sequencing (scRNA-seq) of CD7N CAR-T cells after tumor exposure revealed a unique AQP3+ proliferative CD4+ T cell subset, characterized by broad activation of Src/Ras/ERK and Bcl-2 pathways, ligand-receptor (L-R) interactions of the TNF superfamily, and high expression of SOS1 and KLF2, which could serve as novel modifiers to prevent immune senescence and terminal exhaustion.^2^

### Conclusion

CAR-T therapy is promising for T cell malignancies; however, its full potential has yet to be fulfilled, as major challenges remain. The strategies utilized in our recent study addressed these barriers collectively, paving a way forward for the clinical development of safer and more effective CAR-T for T-lineage neoplasms. As CD7 is expressed in subsets of acute myeloid leukemia and in some non-hematologic solid tumors, and CCR4 is expressed by tumor-infiltrating T-regulatory cells,^6^ future investigations of the CCR4/CD7 CAR in myeloid malignancies and solid tumors are warranted.^10^

## Acknowledgments

This study was partially supported by the General Research Fund (17122923; 17103322), Collaborative Research Fund (C4008-23 W), Research Grants Council of Hong Kong, Hong Kong SAR, China, and by research funds from the GOH Foundation, Singapore.

## Declaration of interests

The authors declare no conflicts of interest.
